# Endoscopic biliary ethanol ablation using a novel multi-hole balloon catheter: In vivo feasibility study in a swine model

**DOI:** 10.1371/journal.pone.0283733

**Published:** 2023-03-31

**Authors:** Tadahisa Inoue, Hiromu Kutsumi, Mayu Ibusuki, Masashi Yoneda

**Affiliations:** 1 Department of Gastroenterology, Aichi Medical University, Nagakute, Aichi, Japan; 2 Center for Clinical Research and Advance Medicine, Shiga University of Medical Science, Seta Tsukinowa-cho, Otsu, Shiga, Japan; Shanghai Jiao Tong University Medical School Affiliated Ruijin Hospital, CHINA

## Abstract

**Background:**

Minimally invasive local treatment could be a good option for the treatment of extrahepatic cholangiocarcinoma (eCCA). This study aimed to evaluate the feasibility of a novel local treatment method, endoscopic biliary ethanol ablation (EA), in vivo using a swine model.

**Methods:**

This study utilized a prototype of the newly developed multi-hole balloon catheter. The swine bile duct was ablated using this balloon via the same approach as the conventional endoscopic retrograde cholangiography procedure. The study outcomes included technical success, clinical success, and adverse events associated with endoscopic biliary EA.

**Results:**

Fourteen miniature pigs underwent endoscopic biliary EA. Technical success was achieved for all endoscopic EA procedures without any hindrance. All pigs were reared and followed up for a median 35-day period after the procedure. No change was observed in the bile duct wall in one case, in which sufficient contact was not achieved between the balloon and bile duct wall. Except for this case, stricture formation occurred at the site of ablation, where the epithelium was sloughed and necrosis with denaturation replaced the granulation tissue and fibrotic changes. The median length and depth of the ablation area were 17.05 and 2.21 mm, respectively. No adverse events were observed, except for the formation of bile duct strictures and sequelae associated with strictures.

**Conclusions:**

This preliminary study was the first to report endoscopic biliary EA using a novel multi-hole balloon catheter, which demonstrated technical feasibility and potential for the treatment for eCCA.

## Introduction

The prognosis of extrahepatic cholangiocarcinoma (eCCA) is poorer than that of most other malignancies [1,2], necessitating improvements in its treatment outcomes. Surgical resection is the only curative treatment modality for eCCA; however, surgery is contraindicated at diagnosis in several cases due to metastasis and/or local advancement. Although systemic chemotherapy, such as gemcitabine plus cisplatin regimen, is the first line of treatment in unresectable cases, its therapeutic effect is insufficient because the median overall survival duration with this regimen is reported to be approximately one year [3,4]. Furthermore, even if the lesion is resectable, highly invasive surgical procedures such as hepatectomy and pancreaticoduodenectomy are required frequently. eCCA is likely to occur in the elderly, in whom highly invasive surgery is difficult because of advanced age and/or underlying disease. Moreover, chemotherapy also tends to be contraindicated in patients who are poor surgical candidates.

In this milieu, minimally invasive local treatment methods including photodynamic therapy (PDT) and thermal radiofrequency ablation (RFA) that directly approach the cancerous lesions from within the bile duct via the endoscopic or percutaneous approach have garnered attention in recent years [5]. Although the potential of these treatment modalities is certain, no study has established their safety and utility as standard local treatment options for eCCA. Therefore, we invented a novel local treatment method for eCCA known as endoscopic biliary ethanol ablation (EA). This study aimed to evaluate the feasibility of this novel EA procedure in vivo using a swine model.

## Materials and methods

### Ethanol ablation catheter

**Fig 1:**
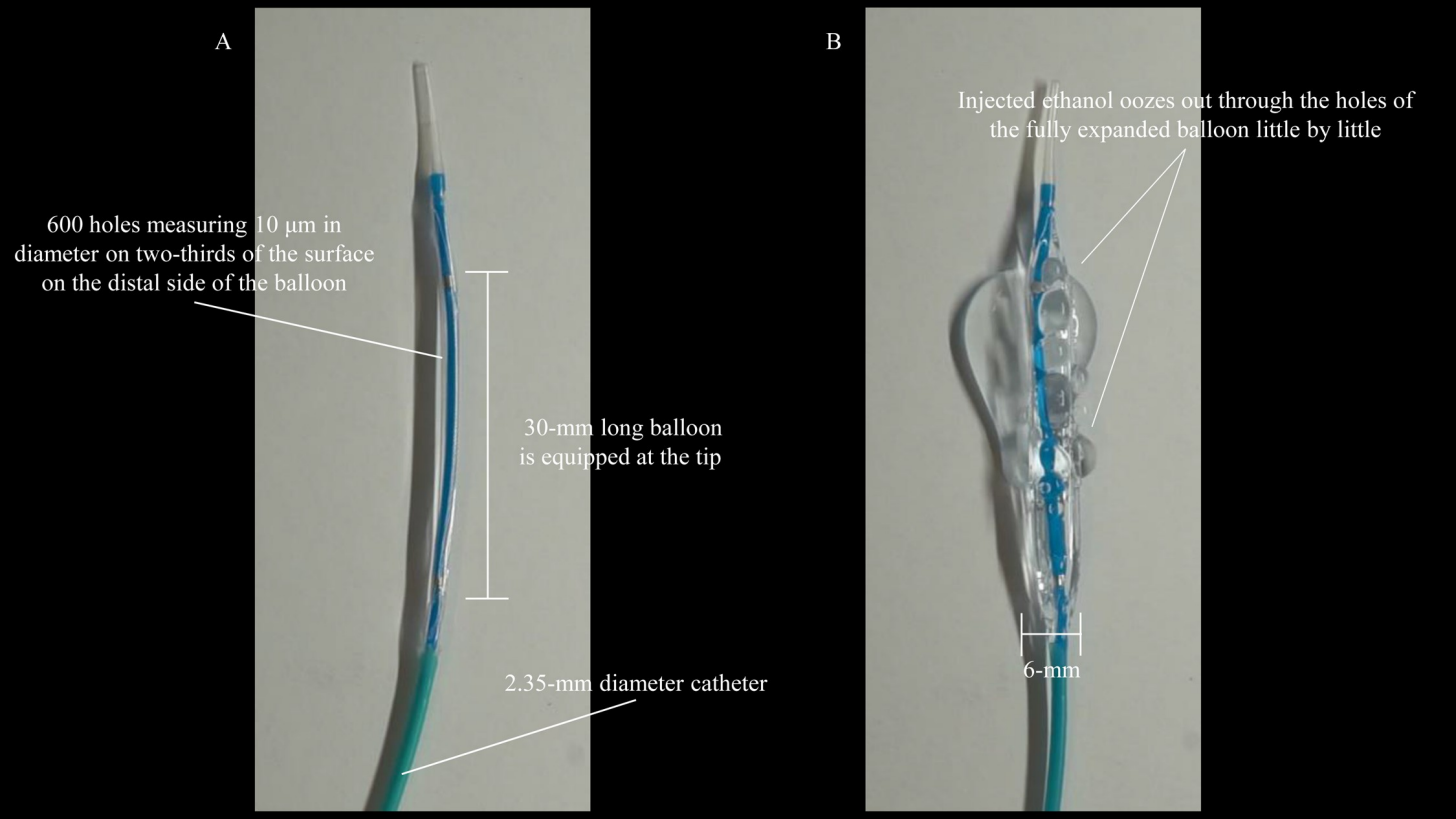
Prototype of the newly developed multi-hole balloon catheter equipped with a 30-mm long balloon of 6, 8, or 10 mm diameter, which has 600 holes of 10 μm diameter on two-thirds of the surface on the distal side of the balloon (**A**). When ethanol is injected into the balloon, the balloon expands initially until the full size is achieved; subsequently, a small amount of ethanol gradually oozes out of the balloon through the holes (**B**).

This study utilized a prototype of the newly developed multi-hole balloon catheter (Japan Lifeline Co., Ltd., Tokyo, Japan). The catheter measures 2.35 mm in diameter and 2500 mm in length and is equipped with a 30-mm long balloon at the tip, with diameters of 6, 8, or 10 mm. Six hundred holes measuring 10 μm in diameter were created by laser on two-thirds of the surface on the distal side of the balloon at regular intervals. When ethanol is first injected into the balloon, the balloon initially expands until the full size is achieved; subsequently, little amounts of ethanol gradually ooze out of the balloon through the holes (Fig 1 and S1 Video).

### Experimental procedure

The Institutional Animal Care and Use Committee of Aichi Medical University approved this study. The study was carried out in compliance with all relevant guidelines. Additionally, the ARRIVE guidelines have been followed for the study. Fourteen miniature pigs with a body weight of approximately 30 kg were used for this in vivo experimental study. They were premedicated with intramuscular injection of ketamine (10 mg/kg), xylazine (2 mg/kg), and atropine sulfate (0.5 mg). Subsequently, general anesthesia was administered and maintained using isoflurane (1%–3%).

The pigs were maintained in the supine or prone position after general anesthesia administration. The TJF-240 duodenoscope (Olympus Medical Systems, Tokyo, Japan) was inserted until the duodenal bulb, and biliary cannulation was conducted with a standard catheter and 0.025-inch guidewire. Cholangiography was performed, followed by the insertion of a multi-hole balloon catheter over the guidewire into the bile duct through the working channel of the scope. The diameter of the balloon was determined based on the thickness of the bile duct on cholangiography. The balloon was located at the target position, followed by inflation of the balloon with absolute ethanol injection; subsequently, an assistant gradually injected another 3 mL of ethanol into the balloon catheter over 60 s. Ethanol permeated the bile duct wall in contact with the balloon. The balloon was deflated after ethanol injection, the residual ethanol was aspirated, and the catheter was removed.

The pigs were reared for 1–2 months after the procedure, followed by endoscopic retrograde cholangiography evaluation in the same manner as the initial procedure. Moreover, the site of ablation was assessed using cholangioscopy. After the evaluation, the pigs were euthanized and autopsied with an intravenous infection of potassium chloride under general anesthesia. The bile duct and surrounding organs were subsequently removed en bloc. The bile duct was dissected longitudinally to expose the mucosal surface, which was evaluated macroscopically. The resected ablated region and adjacent tissue were fixed in formalin, embedded in paraffin, and sectioned for histological analysis, which entailed staining with hematoxylin and eosin and Masson’s trichrome.

### Outcomes

The study outcomes included technical success, clinical success, and adverse events associated with endoscopic biliary EA. Technical success was defined as the technical success of the procedure from biliary cannulation to the end of EA. Clinical success was defined as that histological effect obtained at the site of ablation. Adverse events were assessed using cholangiography in conjunction with cholangioscopy and macroscopic and microscopic examination findings.

## Results

Details regarding the procedures and outcomes are presented in Table 1. Technical success was achieved for all endoscopic EA procedures without any hindrance. The technical success rate was 100% (14/14). EA was performed for the superior, middle, and lower bile ducts in 8, 4, and 2 subjects, respectively. The 6-mm diameter balloon was chosen in 7 cases, 8 mm in 5 cases, and 10 mm in 2 cases; however, in one case, close contact could not be achieved between the selected balloon and the bile duct wall because the diameter of the bile duct was large. No early procedure-related adverse events were observed.

**Table 1:** Details and outcomes of endoscopic biliary ethanol ablation.

Number of subjects, n		14
Bile duct site ablated, n (%)		
	Superior	8 (57.1)
	Middle	4 (28.6)
	Inferior	2 (14.3)
Diameter of the balloon used, n (%)		
	6 mm	7 (50.0)
	8 mm	5 (35.7)
	10 mm	2 (14.3)
Median rearing period after ethanol ablation, days (range)		35 (29–77)
Technical success, n (%)		14 (100)
Clinical success, n (%)		13 (92.9)
Median length of ablation area, mm (range)		17.05 (12.16–20.55)
Median depth of ablation area, mm (range)		2.21 (1.54–3.01)
Adverse events other than biliary stricture and sequelae associated with the stricture, n (%)		0

**Fig 2:**
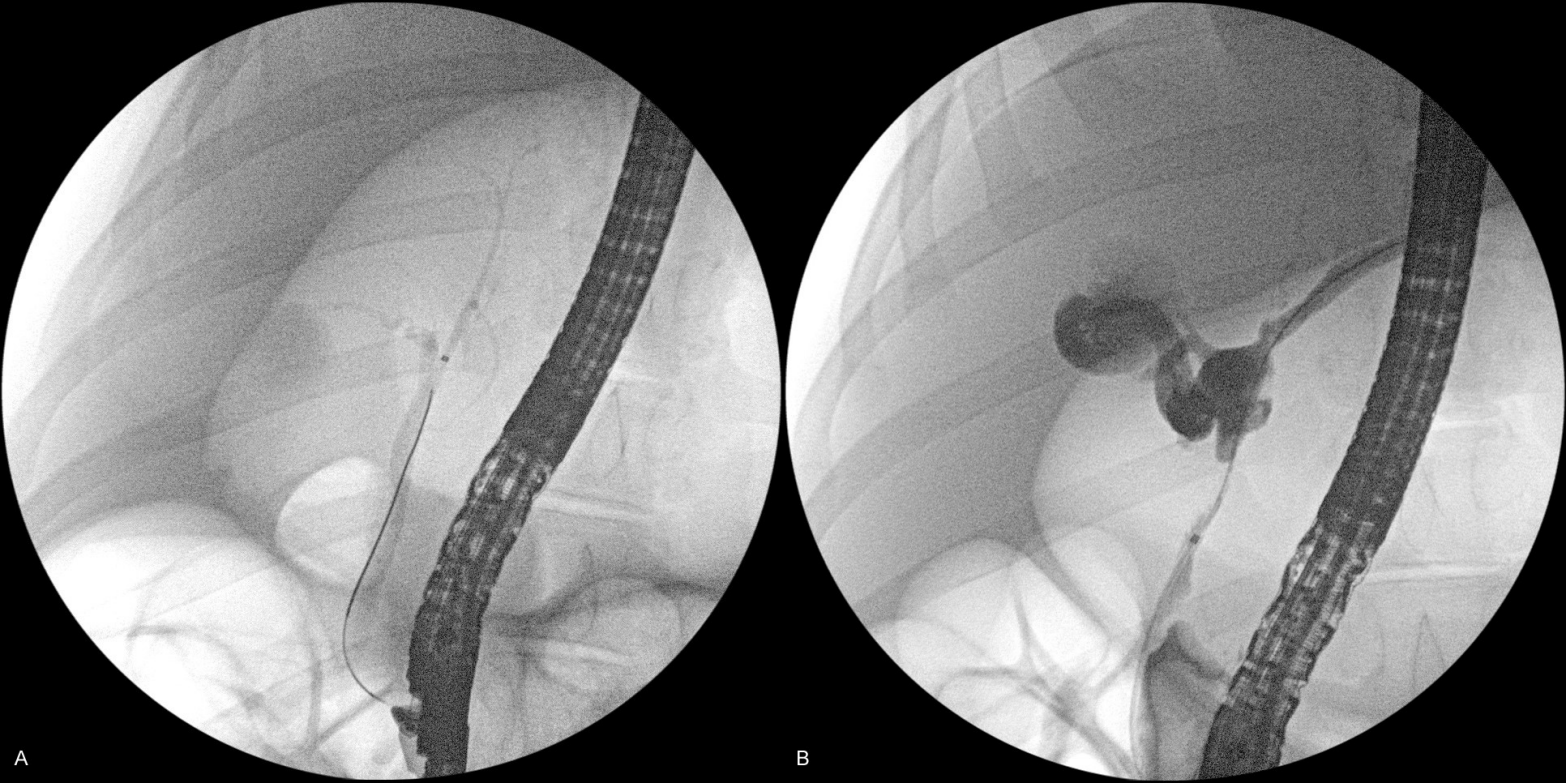
Endoscopic retrograde cholangiography images obtained before (**A**) and 35 days after (**B**) ethanol ablation. Stricture formation is observed at the ablated site, and dilation of the intrahepatic bile duct is observed 35 days after ablation.

**Fig 3:**
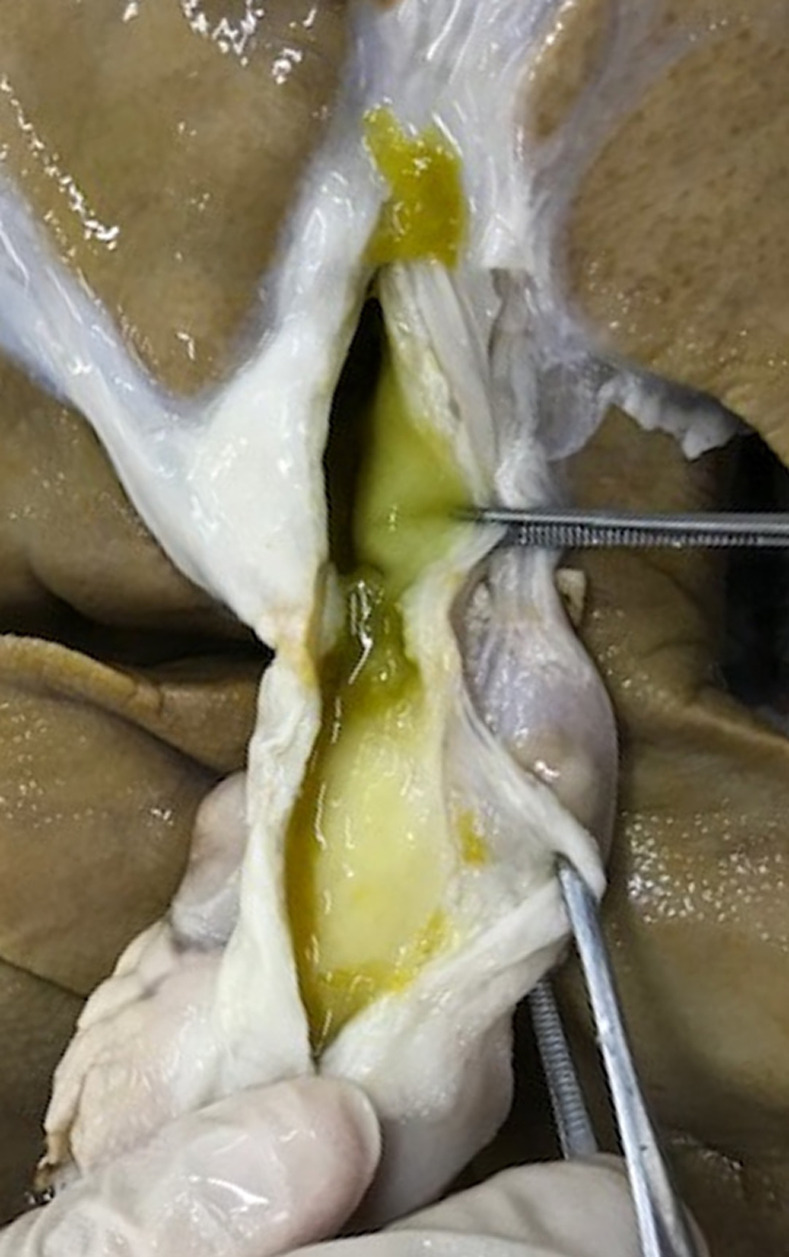
The mucosa at the site of ablation shows the formation of scar stricture on macroscopic examination 35 days after ethanol ablation.

**Fig 4:**
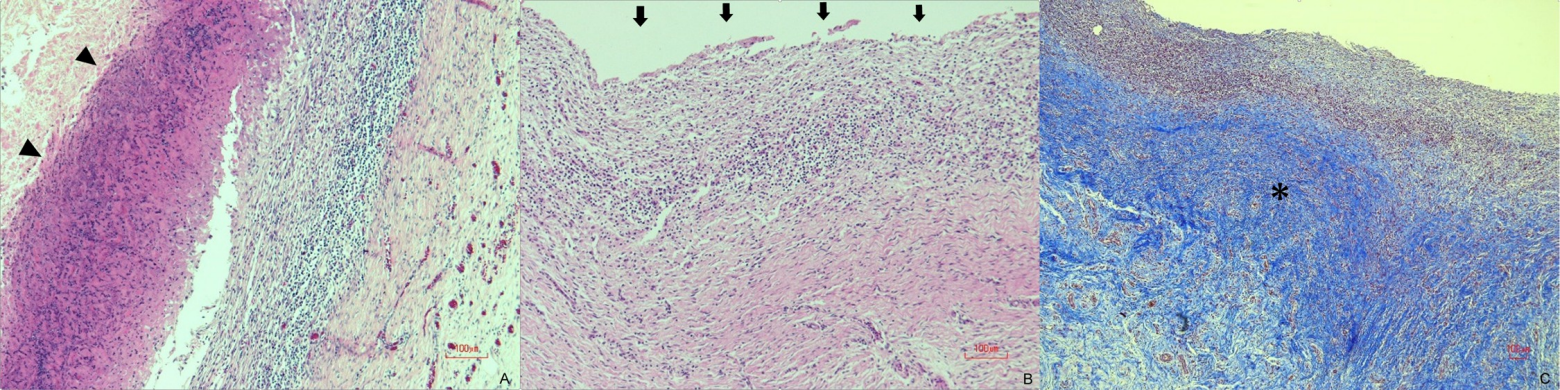
Microscopic examination shows epithelial sloughing (arrow) and necrosis with denaturation (arrowheads) at the site of ablation, which has replaced the granulation tissue and fibrotic changes (asterisk); **A**, **B**, hematoxylin & eosin; **C**, Masson’s trichrome.

All pigs were reared and followed up for a median period of 35 days after the procedure, followed by endoscopic retrograde cholangiography and cholangioscopy, which were conducted to evaluate the site of ablation. No change was observed in the bile duct wall in one case, where sufficient contact could not be obtained between the balloon and bile duct wall. The site of ablation exhibited stricture formation in all cases (Figs 2 and 3 and S2 Video), with the exception of this case. Histological analysis of the samples revealed that epithelial sloughing and necrosis with denaturation had been achieved at the site of ablation, which had replaced the granulation tissue and fibrotic changes (Fig 4), yielding a clinical success rate of 92.9% (13/14). The median length and depth of the ablation area were 17.05 and 2.21 mm, respectively. No late adverse events were observed, except for the formation of bile duct strictures and sequelae associated with the stricture.

## Discussion

This study showed that endoscopic biliary EA using the novel multi-hole balloon catheter was a feasible and technically simple procedure, which yielded a robust histological effect.

eCCA may be unresectable even in the absence of metastasis, since this lesion is prone to extensive intraepithelial spread and progression. Moreover, some patients cannot withstand surgery even if it is deemed possible, because extensive resection involving the liver and pancreas is often required for the curative treatment of eCCA. If local treatment succeeds in curing eCCA or prolonging the prognosis, it will become an important treatment option, especially for such patients, which will represent a watershed moment for the treatment of eCCA.

Studies have reported that PDT can be employed as a local treatment modality for eCCA, and some have already shown that PDT can prolong survival for eCCA [6–9]. However, currently, the use of PDT is restricted and has some limitations. This is because PDT is a complex, time-consuming, and expensive procedure that requires highly specialized devices. Further, photosensitivity is a characteristic and important adverse effect of PDT. Overcoming these issues are the challenges faced by PDT, while robust evidence supporting the utility of PDT for eCCA is needed.

RFA is another local treatment option for eCCA. Although a clear consensus remains to be established, some studies have shown that RFA can prolong survival and biliary stent patency in patients with eCCA [10–12]. Although RFA is technically easier than PDT, patient selection is an issue arising from the current RFA catheter design. The RFA catheter is a bipolar device with two ring electrodes located at the tip of the catheter. A sufficient ablation effect cannot be obtained in cases such as those with short-length strictures, and the electrode contact area tends cause strong burns, which leads to ununiform ablation depth and area [13]. Although a recently developed catheter with smaller ring electrodes and a shorter distance between the electrodes or balloon-based RFA catheter may mitigate this limitation [14–16], this aspect requires further research and improvement.

Biliary EA offers greater technically simplicity, requires a shorter operative time than PDT and RFA, and does not require specialized equipment such as a generator, making the procedure extremely cost effective. The utility of ethanol ablation/injection has already been established for other diseases, including hepatocellular carcinoma [17]. Ethanol is also reportedly cytotoxic to the bile duct wall, since it causes membrane lysis and protein denaturation [18,19]. However, the method of ethanol administration and delivery method presents a hurdle while targeting the eCCA lesion. Therefore, we devised the multi-hole balloon catheter to overcome this issue, which is expected to allow the ethanol to permeate the bile duct wall followed by obtaining adequate ablation depth by adhering the balloon to the bile duct wall. This study constituted the first step in confirming the ablation effect in the area of the bile duct in contact with the balloon. However, it is necessary to conduct a more detailed evaluation of whether the ablation depth can be controlled by adjusting the amount of ethanol in further studies. Whether the intended ablation range can be obtained or not is a vital requirement of local treatment. Additionally, further detailed examination of the safety of this procedure is necessary, since a certain amount of injected ethanol can flow into the intestinal tract. Occluding the duodenal papilla side with another balloon may offer a better solution to prevent the flow of ethanol into the intestinal tract while performing EA [19].

The results of this study should be considered in the context of its limitations, which arise from its in vivo design that used a normal swine bile duct for the experimental procedures. Differences exist between healthy and cancerous tissues and between human and pig tissues; thus, it is still uncertain if the treatment is clinically effective for eCCA. Additionally, the adverse events after EA could not be evaluated with respect to the subjective symptoms such as abdominal pain and nausea. Cholangitis, jaundice, and abnormal liver function were also not evaluated because regular blood tests were not conducted.

Despite these limitations, this is the first study to report endoscopic biliary EA using the novel multi-hole balloon catheter, which demonstrated technical feasibility and potential for the treatment for eCCA. This preliminary in vivo study could be useful and encourage further investigations into this procedure.

## Supporting information

S1 Video Bench test of the novel multi-hole balloon catheter. (MPG)

S2 Video Cholangioscopic view before and 35 days after ethanol ablation in the swine model. (MPG)
